# Immersive Virtual Reality to Distract From Pain in Children Treated With L-asparaginase by Intramuscular Injection

**DOI:** 10.7759/cureus.34317

**Published:** 2023-01-28

**Authors:** Chia-Chi Chiu, Yu-Ting Lin, Yean Wang, Tsung-Yen Chang, Yu-Chuan Wen, Yi-Wen Hsiao, Shih-Hsiang Chen, Tang-Her Jaing

**Affiliations:** 1 Nursing, Chang Gung Memorial Hospital, Taoyuan, TWN; 2 College of Medicine, Chang Gung University, Taoyuan, TWN; 3 Pediatric hematology and Oncology, Chang Gung Memorial Hospital, Taoyuan, TWN; 4 Pediatric Hematology and Oncology, Chang Gung Memorial Hospital, Taoyuan, TWN

**Keywords:** l-asparaginase injections, acute lymphoblastic leukemia, nursing, pain, children, virtual reality

## Abstract

Background

Treatment-related pain and discomfort are two of the most common manifestations in children with acute lymphoblastic leukemia (ALL). Patients with ALL are usually treated with L-asparaginase (L-ASP) by intramuscular injection. Children receiving L-ASP chemotherapy must bear adverse reactions such as pain caused by intramuscular injections. The use of virtual reality (VR) distraction technology could be a non-pharmacological intervention to bolster patients’ comfort and decrease anxiety and procedure-related pain within hospital settings.

Methodology

The study explored the potential benefits of VR as a psychological intervention to induce positive emotions and reduce pain levels in participants receiving L-ASP injections. Participants in the study had the opportunity to select a nature theme of their choosing during their treatment session. The study provided a noninvasive solution that promoted relaxation to reduce anxiety by shifting an individual’s mood positively during treatment. The objective was met by measuring participants' mood and pain levels before and after the VR experience and participant satisfaction with the use of the technology. This mixed-methods study of children aged six to 18 received L-ASP between April 2021 and March 2022, using a Numerical Rating Scale (NRS) with sheer numbers ranging from 0 (no pain) to 10 (extreme or most pain possible). Semi-structured interviews were conducted to collect new data and explore participants’ thoughts and beliefs about a particular topic. A total of 14 patients participated. Descriptive statistics and content analysis are used to describe the data analyzed. VR is an enjoyable distraction intervention for managing treatment-related pain in ALL with intramuscular chemotherapy.

Results

Eight of 14 patients found a reduction in perceived pain after wearing VR. During the intervention implementation, the primary caregivers felt that the patient's pain perception was more positive when using the virtual reality device, and there was less resistance and less crying.

Conclusions

This study describes changes and experiences associated with pain and physical discomfort in children with ALL receiving intramuscular chemotherapy. This teaching model is applied to developing medical personnel, providing information about the disease and daily care, and educating the participants' family members. This study may expand the usage of VR applications so that more patients can benefit from them.

## Introduction

It is still doubtful that virtual reality (VR) effectively reduces pain for pediatric patients [1]. Pharmacological treatments for pain are full-fledged in the emergency department, where pain management strategies include opioid analgesics and benzodiazepines [2]. Pharmacological treatment could be effective when the patient feels comfortable and has no side effects [3]. High levels of sedation may lead to increased mortality and length of stay [4]. VR immersion during intramuscular injection may lead to reductions in pain perception. Several studies have investigated VR (e.g., algology, oncology, and anesthesia) to reduce pain and improve patient comfort [5-8].

The experience of pain is common in pediatric care, especially among children receiving oncology care and treatment. According to the Ministry of Health and Welfare statistics of the Executive Yuan in 2019, the major cause of death for teenagers aged 12 to 18 was malignancies, accounting for 16.2%. Acute lymphoblastic leukemia (ALL) is the most frequent childhood malignancy, accounting for 25% of all childhood cancers [9]. In 90% of the patients with ALL, L-ASP has achieved significant improvement in therapy outcomes and complete remission. They must bear the adverse events, such as pain and anxiety, caused by the injection. It can be observed that the pain degree of receiving an L-ASP injection is much greater than that of other intramuscular injection drugs [10]. Patients of all ages experience fear, withdrawal, and crying when receiving an intramuscular injection of L-asparaginase, disputes with family members, and even beleaguered treatment from medical staff.

With the rapid development of information technology, VR, augmented reality (AR), and mixed reality (MR) applications are becoming more extensive. The medical care field is gradually introducing VR, AR, and MR technology. Use computer graphics and related hardware to generate a three-dimensional virtual environment and interact with virtual objects through various sensing devices such as head-mounted displays, stereo glasses, or 3D mice. Real-world-like visual, auditory, and other sensory simulations reduce visual and auditory sensitivity to the surrounding environment. The subjects can concentrate, immerse themselves in VR, and temporarily detach from the real world by shifting their attention. As a distraction technique, it can effectively reduce the fear of children in the interventional procedure, including lumbar puncture [11,12]. Studies have shown that VR has been used as an additional tool for treating acute and chronic pain and can effectively reduce the pain caused by injections and dressing changes [13]. However, a few pieces of literature point to the effectiveness of chemotherapy drugs.

The project aims to study the impact of VR on individual perception and sensation in patients receiving intramuscular injections. The primary outcome included the assessment of pain levels before and after the intramuscular injections at the index follow-up visits.

## Materials and methods

Design constraints

Briefly, the study was a single-center, open-label, nonrandomized study with two parts: part A was to evaluate the numerical rating scale (NRS) to assess the pain level after the intramuscular administration of L-ASP, and part B was NRS compared to another session with VR in the same patient. We evaluated the feasibility of VR, which improves tolerance to L-ASP injections. The Chang Gung Memorial Hospital Institutional Review Board approved this study. Written informed consent was provided by patients and/or parents/legal guardians. As this was a descriptive study, the sample size was not estimated.

**Figure 1:**
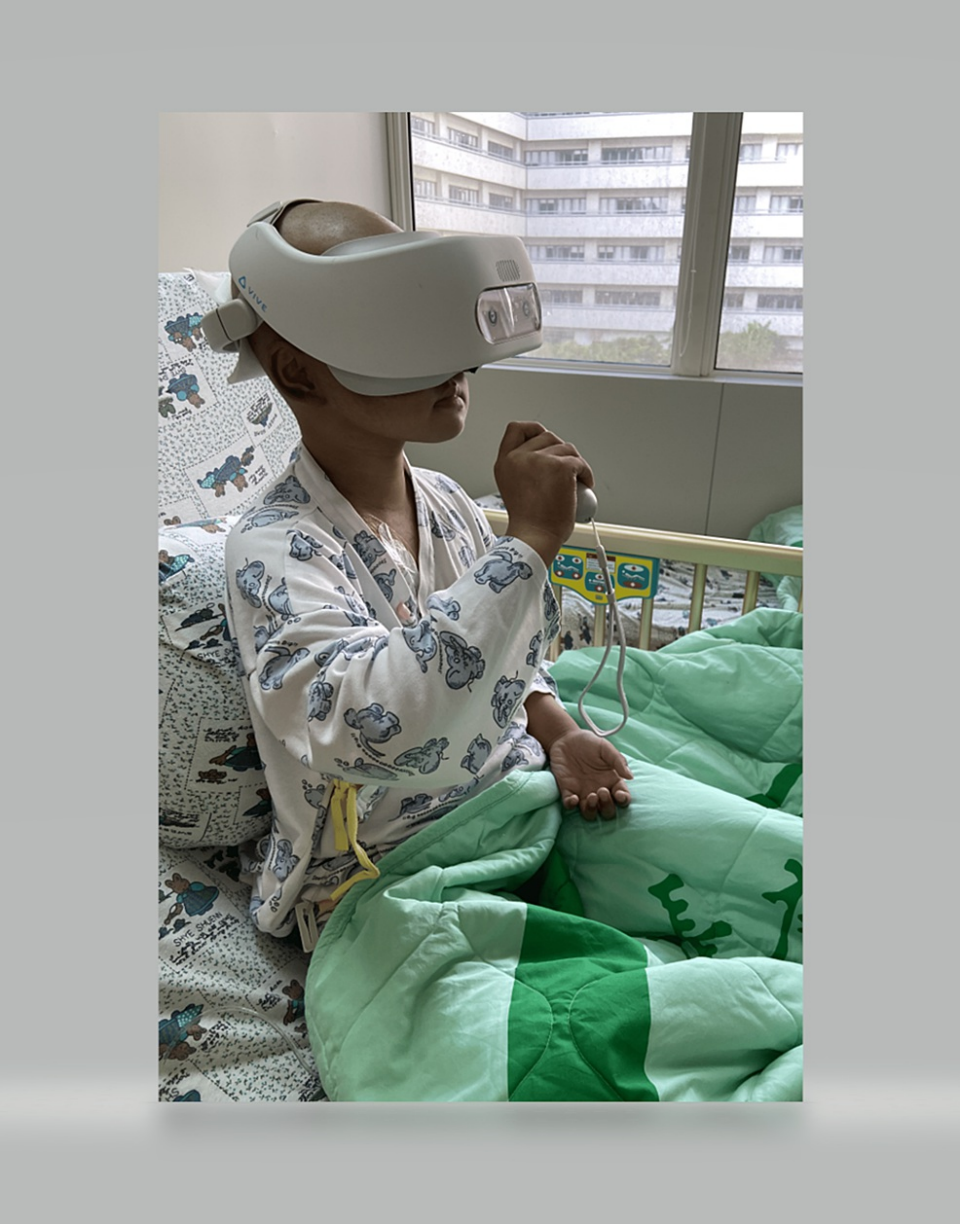
Photo of the participant using the virtual reality application The VR setup and activity to the outside observer

When the VR session started, the equipment and contents of the visual experience were explained to the participants, and questions were answered. They were asked about their pain level on the 0-10 NRS. The participant donned the VR headset, and headphones were placed on the subject. They were given a mouse on a clipboard placed in their lap. The participant then engaged in the VR experience for 20 minutes. Figure 1 shows the VR setup and activity for the outside observer.

**Figure 2:**
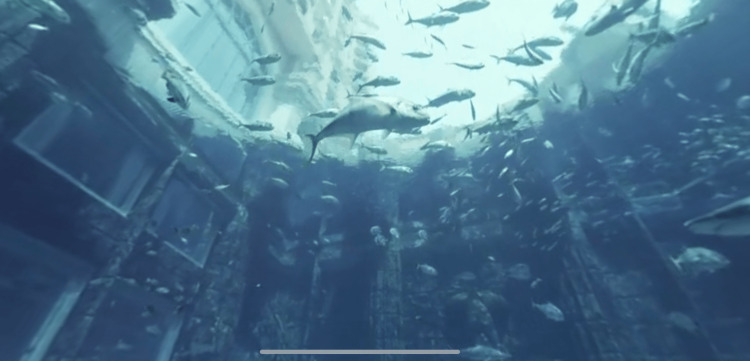
Virtual reality experiences bring aquarium audiences closer to nature without captivity The VR application is an immersive journey that integrates the 360˚ VR video distribution landscape. Participants are taken on a tour through a virtual landscape.

The VR application is an immersive journey that integrates the 360˚ VR video distribution landscape. Participants are taken on a tour through a virtual landscape (see Figure 2 for screenshots). Objects include forests, hills, snow scenes, aquariums, and dinosaurs. The speed is steady and cannot be manipulated by the participant. The applicant delivers ambient music through headphones. The music is lowered in volume, and participants hear the experimenter and converse as much as they desire. Once started, the participant “travels” through the landscape at constant speed until the application automatically stops at the time limit (20 minutes). The scene is 360˚, and participants can seamlessly look forward, left, right, above, below, and behind them. The participant can interact with various aspects of the landscape as they travel using the buttons of a mouse.

Sample, setting, and data collection

All patients with ALL who were undergoing this treatment plan were eligible for VR. Patients should be awake and cooperative, but an explanation of the elements of the procedure remains necessary. We also choose a lightweight VR headset that can be worn comfortably for at least a few hours and is made of soft and durable materials.

Dialog with patients and parents was shown to be critical for informed decision-making. Most children older than five years of age can provide meaningful self-reports of pain intensity if they are provided with age-appropriate tools and training [14]. Discussion should provide complete and understandable information on experimental strategies and emphasize the hope for parenthood as a positive decisional factor.

Data analysis

This study was mixed-methods research. The researcher and the medical care team of the Children’s Hematology and Oncology Department collaboratively formulated a research design and executed it. The evaluation was performed before the individual case had the chemotherapy drug injection. During the patients with ALL receiving an intramuscular injection of L-ASP, the same patients were divided into self-control at different time points. Each patient received two assessments during chemotherapy. Patients in the first assessment did not use VR and in the second assessment participated in a VR intervention during treatment.

In contrast, the second time was with the VR equipment, wearing the VR at least 20 minutes before and during chemotherapy. Physiological assessment includes: changing vital signs (increased heart rate, increased blood pressure, increased or decreased respiratory rate), cold sweat, immobilization, flinching, or moaning when touching the injection site. Vital signs information was documented in the electronic health record at Chang Gung Memorial Hospital. These physiological assessments were a reference for the pain assessment. Moreover, the patient's pain degree was recorded using the NRS. Interviews were conducted with children and their primary caregivers, thus allowing participants to share their broader viewpoint of pain and anxiety experiences. The two-tailed t-test was used to detect the effect from both directions.

## Results

The study included 14 children, and eight (57%) patients had greater pain reduction compared with the first assessment. The reduction tended to be more evident after wearing VR headsets. The pain index significantly decreased for the pain assessment results of these two trials. It was found that the two-tailed P-value was 0.0048 as presented in Table 1, which was considered statistically significant. All interviews were then conducted and eight of them reported a reduction in NRS pain intensity. The investigators found that NRS scores were statistically lower when patients were immersed in VR. The results suggested that VR interventions for pain reduction tended to be more efficacious for adolescents aged 10-17 years than for younger children (P = 0.218).

**Table 1:** Numerical rating scale assessed before and after the VR intervention The P-value was 0.0048 Abbreviations: NA, not applicable; NRS, numerical rating scale; VR. virtual reality

No	Age in yrs	NRS without VR	NRS with VR	Interview content
1	9	8	NA	Refusal to put on VR headset at the time of injection
2	17	9	2	Marked reduction in perceived pain
3	8	10	9	Mild reduction in perceived pain
4	6	9	4	Marked reduction in perceived pain
5	9	10	6	Marked reduction in perceived pain
6	15	10	1-2	Mild reduction in perceived pain
7	6	10	NA	Refusal to put on VR headset at the time of injection
8	17	3-4	1-2	Marked reduction in perceived pain
9	15	10	8	Mild reduction in perceived pain
10	8	10	NA	Refusal to put on VR headset at the time of injection
11	17	5	NA	“I feel dizziness while using a VR headset.”
12	15	8	8	No effect on perceived pain
13	12	8	6	Mild reduction in perceived pain
14	13	7	NA	“I feel dizziness while using a VR headset.”

The research results showed that the children's pain with VR was not aggravated when receiving intramuscular chemotherapy, and the treatment time was short. Compared with the children without wearing the VR, the children who wore the VR were easier to cooperate with the treatment. Besides, the treatment made it hard to leave a negative impression. The results also revealed that children's pain was relieved after using the VR. During the intervention's implementation, the children's primary caregivers felt that the children’s pain performance was more positive than during previous treatment when using the VR. Furthermore, there was less resistance and crying during the treatment.

Two participants felt dizzy after wearing the VR headsets, so the trial was halted. In addition, three participants expressed their willingness to look at the needle at the moment of injection; thus, the VR was removed. One child had the same pain assessment score without and with the VR. One mother said her daughter felt discomfort, restlessness, and diaphoresis before the injection. After using these, the anxiety and tension of the child before the injection were significantly relieved. In addition, the child was attentive to the videos on the VR.

## Discussion

Procedural sedation and analgesia are appropriate techniques using short-acting analgesics and sedative medicines to facilitate adequate performance. VR has tried to reduce the pain associated with many known medical procedures [15]. In clinical and experimental studies, participants in the VR experience reduced pain and general distress/unpleasantness and reported a desire to provide personalized approaches to VR during painful medical procedures. The Verbal NRS is a widely used self-rated measure of pain intensity. Construct (convergent and known-groups) validity, responsivity, and reliability of the Verbal NRS were vital for children aged six to 17 years [16]. VR is an innovative distraction intervention for reducing the pain of ALL children who need to receive intramuscular chemotherapy. A larger reduction of pain was observed among adolescents who used VR distraction compared with younger children. Thus, age could be a confounding variable and influence the outcome of this study. Moreover, it is a safer intervention than pain medication.

We hypothesize that VR may masquerade as non-pharmacologic analgesia by exerting emotional evaluative processes on the body’s entangled pain modulation system. Parents who believe in scientific and technological progress were more likely to encourage their children to participate in the intervention. This belief changes the assumed balance of harms and benefits. While the underlying neurobiological mechanisms behind VR’s action remain ambiguous, investigations are ongoing to examine the intricate interplay of cortical activation associated with immersive VR. New applications, including VR, have been developed to unleash or enhance evidence-based interventions, such as hypnosis and biofeedback, to treat chronic pain [17]. This study provides real-world outcomes from VR data, exploring its clinical and experimental applications for acute and chronic pain management, focusing explicitly on current trends and developments [18]. Besides, we propose a mechanistic study highlighting VR distraction and neurobiological explanations and sparked a new interest in VR research, implications, and clinical significance. Moreover, Hoffman and his colleagues designed a series of distraction-based VR studies in which 50% of patients reported reduced perceived pain [19].

VR is currently used in various industries and for different purposes. In addition to the use in this article to relieve pain by intramuscular injection, it is also used abroad when children receive vaccine injections. The monsters in the VR games attack the children to divert their attention and relieve pain. The VR content used in this article is all kinds of games and pictures with music and sounds. However, there are no pictures specially designed for medical purposes, for instance, the abovementioned monster attacking children in the VR. If the attack screen is with acupuncture simultaneously, the effect may be better. Nevertheless, there are few such designs in Taiwan currently. We hope to find a software development company for collaboratively designing suitable game software in the future.

Sample sparsity did not allow testing for clinical benefits. However, our results prove that VR can achieve a theoretical health education assessment for teaching. The VR model can be provided as a new teaching tool for training medical personnel, children's daily care, and health education. We hope more VR content can be written so that more and more people can benefit from it.

Notwithstanding this, our study is not without limitations. It was conducted in a single center, and the longitudinal cohort had a small sample size. However, young kids usually need to gain the vocabulary to discuss their feelings. Although there is no theoretical practice concerning the minimum age for recruitment, there is agreement that it should be at most six years. All members of the medical team, including physicians, nursing staff, and case managers, were trying to create incentives to enhance care coordination and established a therapeutic, interpersonal relationship. We also encouraged family members and peers to accompany and praise, establish positive self-identity, and assist patients in receiving chemotherapy and physical appearance changes. Research shows that virtual reality reduces children’s pain, fear, and anxiety. Different studies conclude that VR reduced pain in some medical procedures, including access to a venous port, blood draws, intravenous injections, pediatric immunization, children's pulp therapy, and phlebotomy in children [20-25].

Medical staff must face these problems in cancer patients to help them turn these unpleasant treatment processes into positive attitudes, guide them to re-establish self-worth, and hope that this nursing experience can provide a future reference for caring for juvenile cases.

## Conclusions

The logic behind why VR may reduce procedural pain is that pain requires conscious attention. Being immersed in a virtual world subverts the attention, leaving less of it available to process pain signals. This study was only conducted when ALL patients received L-ASP injections. Similarly, VR is an emerging field that may be used to alleviate other potentially pain-evoking procedures such as intravenous cannulation, lumbar puncture, and bone marrow aspiration. Future studies with large sample sizes, a multicenter design, and an external cohort must confirm our findings.
